# Comparison of Protein Content, Availability, and Different Properties of Plant Protein Sources with Their Application in Packaging

**DOI:** 10.3390/polym14051065

**Published:** 2022-03-07

**Authors:** Anupriya Senthilkumaran, Amin Babaei-Ghazvini, Michael T. Nickerson, Bishnu Acharya

**Affiliations:** 1 Department of Chemical and Biological Engineering, University of Saskatchewan, Saskatoon, SK S7N 5A9, Canada; jwy763@mail.usask.ca (A.S.); amin.babaei@usask.ca (A.B.-G.); 2 Department of Food and Bioproduct Sciences, University of Saskatchewan, Saskatoon, SK S7N 5A8, Canada; mtn620@usask.ca

**Keywords:** plant-sourced protein, biodegradable packaging, physicochemical properties, film formation methods

## Abstract

Plant-based proteins are considered to be one of the most promising biodegradable polymers for green packaging materials. Despite this, the practical application of the proteins in the packaging industry on a large scale has yet to be achieved. In the following review, most of the data about plant protein-based packaging materials are presented in two parts. Firstly, the crude protein content of oilseed cakes and meals, cereals, legumes, vegetable waste, fruit waste, and cover crops are indexed, along with the top global producers. In the second part, we present the different production techniques (casting, extrusion, and molding), as well as compositional parameters for the production of bioplastics from the best protein sources including sesame, mung, lentil, pea, soy, peanut, rapeseed, wheat, corn, amaranth, sunflower, rice, sorghum, and cottonseed. The inclusion of these protein sources in packaging applications is also evaluated based on their various properties such as barrier, thermal, and mechanical properties, solubility, surface hydrophobicity, water uptake capacity, and advantages. Having this information could assist the readers in exercising judgement regarding the right source when approving the applications of these proteins as biodegradable packaging material.

## 1. Introduction

There is a trend to replace conventional plastics with biodegradable plastics as a result of changing global conditions, reduced petroleum supplies, and an ever-growing demand for plastics [1]. Bioplastics from renewable raw materials are eco-friendly and more sustainable than petroleum plastics, making them a potentially attractive product for the industry [2]. It has been discovered that bioplastics can be manufactured from starch [3], cellulose [4], proteins [5], and other biomass resources [6]. Hence, there are distinct properties of proteins that make them permissible, unique, and distinguishable from other biodegradable sources.

**Figure 1:**
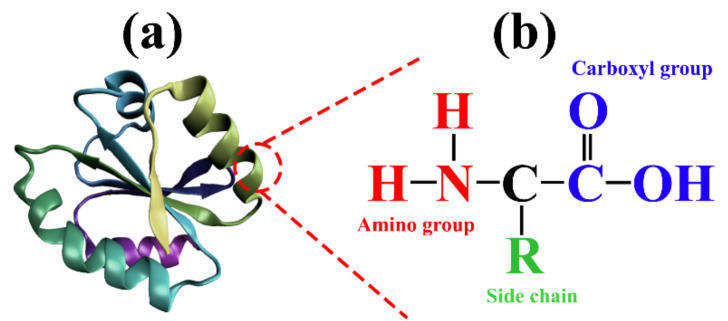
Chemistry of proteins. (**a**) 3D structure of protein molecule, and (**b**) abundance amino acid molecules in the protein polymer.

Proteins (Figure 1) are the naturally occurring macromolecules constructed from long chains of amino acids, composed of carbon, hydrogen, oxygen, nitrogen, and sulfur [7]. All of the proteins are comprised of different arrangements of the same 20 kinds of amino acids [8]. Their structure varies greatly, and the shape is critical to its function. Changes in temperature, pH, and chemical exposure may lead to permanent changes in its shape, leading to a loss of function or denaturation [9].

### 1.1. Opportunities of Protein Sources from Underutilized Biomass for Non-Food Application

Underutilized food crops or agricultural waste by-products are called second-generation feed stock. This biomass has been optimized, allocated, and commercialized for different sectors. The types of industries include chemical industries, biorefineries, and bioplastics, as well as food and the animal feed industry [10]. In addition, the biodiesel production from meals of rapeseed, sunflower, soybean, palm oil, and *Jatropha curcas* was 8.6 Mt in 2007. Biodiesel is also produced from annually harvested crops like rapeseed, sunflower, sugarcane, and many others contributing to energy output. Many cropping residues, vegetable leftovers, agricultural residues, food processing residues, aquaculture residues, and edible and non-edible oilseed cakes with high potential are converted into biofuels, heat, electricity, surfactants, adhesives, polymers, cosmetics, bio compost, biogas, and bio-based green products. This indicates the availability of possible substitutes to be used that are environmentally friendly, add value, and protect the climate [11].

Polyhydroxyalkanoates (PHA) are a potent biological substitute for petrochemical plastics; however, its commercialization is 5–10 times more expensive than petrochemical opponents due to the raw materials amounting to half of its cost [12]. A study performed by replacing the raw material from cheap, renewable agro-industrial by-products like ragi husk and sesame oil cake using enzymatic hydrolysates reduced the cost of production by 40–50% [13]. In some cases, the manufacturing of bioplastics from renewable raw materials requires lower energy than petroleum plastics [14].

Whey and caseinates find their application in the textile, pharmaceutical, and biomedical industries. They have been studied as textile coatings [15], textile dyes [14], and functionalized textiles resulting in enhanced staining, improved abrasion resistance, thermal stability, water-solubility [16], anti-microbial, anti-feeling, and tensile strength functional properties and appropriate technology [17]. Furthermore, caseins for nanofibers have been employed in tissue engineering and whey proteins in nanoparticles due to their biological properties such as encapsulation efficiency, good stability, safety, lack of undesirable side effects, integrity, and drug delivery capabilities [18].

The compulsion towards replacing conventional plastics with the sustainable alternative of bioplastics arises mainly due to rapidly growing environmental concerns [19]. Additionally, there is a high risk of contamination due to the leaching of carcinogenic and toxic chemicals in landfills and groundwater, exertion of hot and toxic gases during incineration, and secondary air pollution due to incomplete combustion [20]. The migration of chemical compounds from food packaging to foods causes physical harm to animals and humans and threatens survival [21]. According to statistics, global plastic production aggregated until 2015 exceeded 8.3 Gt, resulted in 6.3 Gt of plastic waste, and was expected to increase to 12 Gt by 2050 [22,23].

A protein’s natural state can either be fibrous or globular. Those belonging to the former class are insoluble in water and serve as the basic structural elements of animal tissues. Glandular proteins are soluble in water or aqueous solutions of acids, bases, or salts, and are found widely in living organisms. The molecular structure of fibrous proteins is characterized by their extended state and close association with each other in parallel structures, often through hydrogen bonds. In globular proteins, hydrogen, ionic, hydrophobic, and covalent (disulfide) bonds act together to form complicated spherical structures (Figure 1) [24,25]. These proteins display a wide variety of chemical and physical properties, depending upon the relative quantities of amino acid residues and their arrangement along the polymer chain [26]. In the production of edible films, collagen has received the most attention among fibrous proteins. There has been substantial research into the film properties of several globular proteins, including wheat gluten, corn zein, soy protein, whey protein, and mung bean protein [27].

Proteins have been demonstrated to provide high film-forming ability [28], barrier properties for gases [29], adhesion to various substrates [30], good mechanical properties [31], high intermolecular binding [32], and act as fertilizer during the degradation of nitrogen sources [27]. In contrast, these offer diverse opportunities to modify inherent protein properties, which is an excellent tool according to its applications. Physical, chemical, and enzymatic modifications are done to adjust the properties based on the specific demands of the products [33]. Thus, sustainable plant-based proteins can resolve the existing problems.

The usage of proteins in edible packaging is also an advantageous novel substitute for improving product stability [34], quality [35], extending shelf life [36,37], reducing packaging waste [38], and serving as a matrix to incorporate bioactive compounds to enhance the nutritional composition of food [38].

### 1.2. Opportunities of Protein Sources for Edible Bioplastic

The global availability of the valorization of protein fractions reduces the risk of shortages and speculation peaks worldwide, so that food security and market stability are maintained. According to the Food and Agricultural Organization, food waste proteins are increasing and are anticipated to amount to approximately 126 Mt by 2020 [39]. It is promising because of its high protein content, well-balanced amino acid composition, and prior removal of allergic or toxic substances before utilization.

Covering foods with edible films substitutes synthetic packaging, satisfactorily prevents moisture and flavor loss, controls the exchange of gases, increases the shelf life, and transits active substances like antioxidants [40], and has antimicrobial [40], antifungal [41], and other applications [27]. The application of corn zein coatings on tomatoes proved to delay color change, reduce firmness, and lose weight during storage [42]. Many protein isolates from defatted pressed meals (soybean, peanuts) have already been used as emulsifiers, stabilizers, fortifiers, and foaming agents. Films from blends of defatted soy protein, papaya puree, and gelatin improve the mechanical barrier, optical, and structural properties, with highly significant edible film properties [43].

### 1.3. Challenges in the Utilization of Protein Sources

Globally 327,000 tons of green plastics are produced on average and 12.3 Mt are in consumption [44]. A study was performed on existing bioplastics, and 67% of products (including starch- and cellulose-based plastics) were found to contain the same toxicity as conventional plastics due to the variety of chemicals used either as additives or during manufacturing [45]. These limitations need to be identified and overcome. Additionally, the efficient utilization of by-products directly impacts the economy of the country.

Plant protein-based bioplastics are gaining popularity, but there exists a limitation in packaging due to their poor mechanical and surface hydrophobicity properties [46]. In addition, the different protein sequences and functional properties present in different sources require optimized study for bioplastic preparation. Designing exclusive parameters like processing conditions, selection of physical [47,48], chemical [49], enzymatic or cross-linking treatments [50], usage of plasticizers, and blending of other sources [51,52] for the preparation of packaging becomes tedious, increasing the cost of production. The limited studies available for the comparison of the majority of protein sources on their mechanical properties, moisture barrier, thermal properties, solubility, and degradation are additional challenges.

A variety of sources containing a high availability of crude protein are utilized as a protein supplement in poultry and livestock feed and fertilizers due to their high nutritional value and richness in minerals and vitamins. Notably, sources like forage crops, oilseed meals, and sugarcane by-products are preferred as animal feed and have potential competition with non-food applications [53,54].

Optimistic and specific procedures need to be discovered for high protein recovery. The most significant challenges lie in the process of extraction, yield recovery, and the cost-effectiveness of the product to replace existing products. Enzyme-assisted extraction is used to replace harsh chemicals, thus being a green extraction method. Such enzymes rely on operational conditions and environmental factors like pH and temperature [55]. However, in certain cases of protein extraction, the output is irrespective of the type of enzyme being used [56].

Films and coatings are mainly developed from animal and plant proteins like soy, zein, gluten, whey, casein, collagen, and many others. However, susceptibility to the proteolytic enzymes present in foods when proteins are coated or made as edible films and clear label identification are needed due to a high number of individuals prone to allergic reactions to milk fractions, wheat, soybean, and peanuts [57].

Many studies predominantly involve the casting method for the preparation of complex films and cannot be developed on an industrial scale [58]. Rich plant protein sources with their total protein content and with the top global producer countries are listed in Table 1 and Table 2 [59,60,61,62]. Specific data on cover crops in Canada are summarized in Table 3 [63].

## 2. Method for the Formation of Protein-Based Packaging

Different sources of proteins and proteins extracted from the waste by-products being a sustainable ecological burden are utilized in various packaging applications [60]. Biodegradable packages, edible films, edible cutlery, thermoforms, trays, and coatings are also used as wraps, casings, pouches, bags, and covers as an alternative to synthetic and atmospheric packaging [64,65,66,67,68,69,70,71]. According to the protein source, different methods of the formation of plastics are being used. Solution casting is extensively used, as well as extrusion, injection molding, hot press molding, and compression molding. Edible films of all protein sources are generally produced through the solution casting method. According to the literature, bioplastic production from certain sources follows the same method as injection molding for pea, hot-press molding for cottonseed, injection molding for soy, rice and many more [26,72,73]. Flexibility, physical integrity, and mechanical, thermal, and barrier properties are improved by the addition of plasticizers and additives, and through different methods of bioplastics formation [74]. Water [74,75] and different types of polyols like glycerol (GLY), ethylene glycol (EG), diethylene glycol (DEG), triethylene glycol (TEG), tetraethylene glycol, and polyethylene glycol (PEG) are used most frequently as plasticizers in bioplastic development [33,76]. In addition to this, fruit extract, cake oil, disaccharides, algae, and antimicrobial agents are some of the other additives used to improve the physicochemical and functional properties of the bioplastics. Table S1 in the Supplementary Materials section illustrates the type of packaging applications using different plant protein sources, compositions, and methods of formation.

## 3. Comparison of Different Properties of Bioplastics

A comprehensive review on various properties like barrier (oxygen permeability (OP), water vapor permeability (WVP)), thermal, mechanical (tensile strength, elongation at break, Young’s modulus), solubility, and emulsifying properties and surface hydrophobicity is addressed in the Supplementary Materials, Table S2 corresponding to Table S1. Several performed methods like polymeric blending, cross-linking, temperature modifications, pH alteration, plasticization, nanocomposites addition, additives migration, or diffusion to increase the potential by addressing the industrial limitations are also noted [77]. Different properties are focused on depending on the application. For example, moisture barrier properties are concentrated on for the packaging of short shelf-life food products [78,79], whereas mechanical properties are concentrated upon to maintain the physical integrity of the product from external influences [77]. Thermal properties focus on the thermal stability of the packaging on heat fluctuation and the time–temperature characteristics of a product. The solubility of a polymer is analyzed for water-soluble pouches, food coatings, and edible pouches [80]. The wettability of food packaging is essential to safeguard the intended materials from humidity during transportation and storage. It is determined by surface hydrophilicity/hydrophobicity and water contact angle, and in most traditional packages, cellulose and starch possess low wettability properties [81]. It is therefore imperative to analyze each set of properties on the different packing applications mentioned in Table 3 to assess the research gap in plant protein bioplastics.

## 4. Synthesis Methods and Properties of Plant Protein-Based Bioplastics

### 4.1. Comparison of Different Formation Methods in Molding and Extrusion

**Figure 2:**
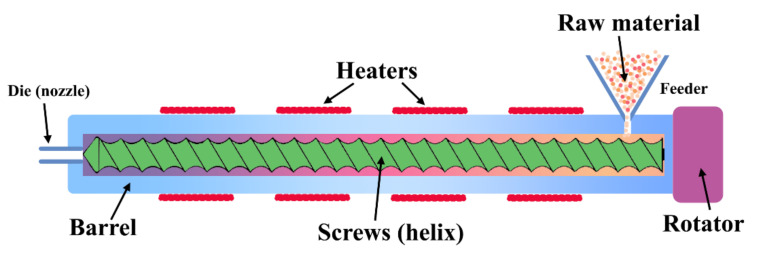
A simple schematic of extrusion processing, demonstrating the transformation of the raw ingredient (such as proteins and other reinforcement materials) to molded composites.

Thermo-molding is an extensively used technology for soy and corn. Casting is the basic form of molding. Compression, injection, and extrusion molding are the other types. This process, with the inclusion of plasticizers, has an impressive tensile strain and good barrier properties though being non-continuous. Extrusion (Figure 2) is intricate because of the complex association/dissociation of proteins under shear and heat treatment. It holds several advantages compared to thermo pressing and wet processing. Influential factors like screw speed, temperature intensity and gradient, presence and amount of additives and plasticizers, and shape and size of die play a crucial role in the denaturation, cross-linking, conformation, and aggregation of proteins. A relatively high temperature is needed to induce these changes in the process of polymer manufacturing [28].

A study on the thermo-molding of pea protein by Perez et al., produced bioplastics with better homogenization, lower energy consumption, intermediate mixing speeds (30 rpm), and shorter mixing times (1 and 10 min) along with good mechanical properties like higher elongation and better tensile parameters. In support of mixing times in the formation of bioplastics by Carvajal et al., high mixing times or speeds were not required to obtain better mechanical and water uptake properties [82].

Jimenez et al., studied soy and found that the increase in mold temperature, post-treatment like dehydrothermal treatment for 12–24 h and ultrasound at 50 kHz for 5 and 45 min to reference systems (injection molding) enhanced mechanical properties. Thermal strengthening gave better mechanical properties in polyethylene or polyvinylchloride, but not as high as in polystyrene. Though ultrasound did not improve these properties, it induced a greater cross-linking degree when performed for 45 min than the reference system by denaturing proteins, leading to weaker bonds. DHT for 24 h also had a higher degree of cross-linking. Future studies are to be conducted on combinations of high temperature and post-treatment [48]. An experiment performed by Gamero et al., showed that an increase in injection mold temperature increased tensile stress and elongation, but no significant difference in Young’s modulus, and hindered water uptake capacity [83].

Compression molding of trays using rapeseed developed by Johansson [58] resulted in an odd smell, large spots, and hardened material. Ball milling the raw material provided the optical difference. Improvisation in the proper mixing of all components is needed for the flow of material. The extrusion of wheat bioplastics has better compatibility than compression molding in terms of mechanical and water uptake properties. An increase in molding temperature beyond 130 °C increased elastic properties and flexural storage modulus with an enhanced microstructure, which was observed in recent studies by Jimenez et al. [84]. This was also supported by Sun et al., who showed an increase in tensile strength, cross-linking density, relaxation time, Young’s modulus, and elongation at break, but showed a drop at the highest temperature when *T* > 65 °C showed a thermo-plastic behavior [85]. In another study, Diani et al., reviewed the influence of die temperature from 85 to 160 °C in sunflower and found that the highest die temperature with a high glycerol, medium water content gave the most regular and smooth film with efficient plasticization. Water was necessary for the extruder’s feeding condition, and glycerol gave a regular flow off the die. Thin, bright, smooth, and drastic mechanical properties were observed for the die at 145 °C. Decreased tensile strength, tensile modulus, and increased elongation were observed at 160 °C [86]. In a study conducted by Alonso et al., the higher molding temperature in rice produced stiffer, compact, and more resistant materials. A more homogeneous structure was obtained at 150 °C, where voids and cracks were absent. Water also acts as a plasticizer along with glycerol, but hinders the processing of samples during the formation of dough [87].

### 4.2. Comparison of Film and Raw Material Treatment Methods

Analyzing different types of film treatment methods helps to compare and determine which is the most appropriate. Choi et al., worked on the heat denaturation of films and reported that this had a significantly positive impact on physical properties, being more stiff and extensible than native pea film [80]. In other research, Liu et al., compared different treatments with peanuts such as heat denaturation of film-forming solution for 30 min from 60–90 °C, ultraviolet irradiation of films for up to 24 h, three ultrasound processes, and chemical treatments with aldehydes and anhydrides. Heat curing was most effective, followed by UV exposure and aldehydes. In the end, peanuts could be used for packaging and other applications with modified treatments giving desirable properties [88].

Regarding the comparison of the treatment methods, Delgado et al., performed pelletizing, sieving, and polycaprolactone addition to rapeseed. They found increased elastic moduli, viscoelastic moduli, and mechanical property of rapeseed, which is com-parable to LDPE polyolefins. These favored a higher degree of homogeneity, and a packed structure was achieved after sieving [89]. Succinylation produces less fragile plastics and improves the color of RPI. A low level of succinylation strengthened the thermal resistance of succinylated rapeseed protein isolate films. Increasing succinylation decreased tensile strength and elongation and increased peroxide oxygen value and WVP. Moreover, 5% succinylated RPI films have the highest elongation and tensile strength, the lowest water vapor permeability and peroxide oxygen value, and are highly hydrophobic compared to other protein edible films like sesame and whey. They have significant potential for use as edible films.

Additional research conducted by Orliac et al., studied the effects of additives on the mechanical properties, hydrophobicity, and water uptake of thermo-molded sunflower films. Fatty alcohols increased surface hydrophobicity and mechanical property and decreased the solubility of the films. Octanoic acid (non-toxic to humans with anti-fungal properties) had the best value for sunflower. Tannins (non-toxic and 100% biodegradable) produced films with lower mechanical properties than aldehydes [90]. Felix et al., interpreted that mixing has a significant influence on rice bioplastics. Optimum mixing conditions of 15 min, 30% glycerol, and the high pressure required are some of the limitations. A lower protein/plasticizer ratio leads to an incompatible system [91]. Similarly, Gómez-Heincke et al., stated that the mixing process hardly induces protein-plasticizer interactions, thus requiring post-thermal treatment at high temperature and pressure to obtain suitable plastics. Potato protein-based bioplastics had a complex modulus value like those found for LDPE. Blends of potato and rice protein in packaging were affected by glycerol content and higher thermal molding temperature treatment [92]. An investigation by Buffo et al., into sorghum revealed that kafirin had the least recovery of 9.57%. Extraction solvents other than ethanol may improve the isolation process, and sulfites may improve yields. They were more intensely colored and have similar WVP to commercial zein films. This can be effectively used as a biopolymer for both edible or non-edible films and coatings [93].

Cottonseed bioplastics, modified by denaturation and cross-linking with urea and aldehydes, improved the thermal stability, water absorption resistance, and mechanical strength due to the Maillard-driven formation of the cross-lined structure. Increasing the plasticizer (glycerol) decreases the denaturation and α-relaxation temperature of CP due to its reduced structural integrity and increased free volume. There is variation in color and odor before and after hot compression molding. It has considerable potential in low-load bearing applications and environment-sensitive industries [73]. Yue et al., commented that different cross-linking agents made a difference in the storage modulus at very low temperatures and had a marginal impact on the creation of different water states. The cross-linked CPBs showed improved thermal stability, water absorption resistance, and mechanical strength [94]. Blends of polycaprolactone and cottonseed protein plasticized with cottonseed oil are suitable in areas of hot melt adhesive. The best plasticizer is found to be cottonseed oil > coconut oil > PEG-400.

### 4.3. Comparison of Mechanical Properties

Mechanical strength depends on the composition and environmental conditions while manufacturing the biopolymer films. It is responsible for maintaining the integrity of the package during handling and storage. The properties include tensile strength, elongation, elastic, and young’s modulus. These may be improvised by different formation conditions, treating parameters, and incorporation of plasticizers according to the sources and applications.

In a study regarding the mechanical properties of packaging material, Sharma et al., optimized sesame film with 9% protein concentration, pH 12, 90 °C, and 10% glycerol. They found that increasing the protein concentration, temperature, and alkaline conditions increased tensile strength, which can be used for packaging or coating applications. The value was better compared to other plant proteins but needs to be improved compared to synthetic films. However, the protein and alkaline pH conditions impacted the optical properties, leading to much less transparent and darker colored protein films [95]. Bourtoom et al., developed a mung protein film produced at pH 9.50, with a heating temperature of 75 °C for 20 min, exhibiting higher mechanical and lower barrier properties. Tensile strength and elongation at break increased as the temperature of the film solution increased from 60 to 80 °C. However, when the pH was increased to 9.5–9.9 under the same conditions mentioned above, the film color was darker [96]. Bamdad et al., found that the intensity of heat treatments and alkaline pH had a significant effect on the physical properties of lentil protein films. It was mentioned that the tensile strength is comparable to conventional polyolefin films, and is lower than soy, whey, and peanut protein. Elongation is also higher than cellophane and other plant proteins mentioned above. A good puncture strength of 1.552 ± 0.2 N for 150 µm film thickness was obtained. Strong and elastic films had medium nutritional value to be used as individual wrappers in large boxes or cartons. Red to brown semi-transparent colored films were used for light-sensitive food packs [97].

Further reading by Choi et al., found that with increasing plasticizer concentration in pea, elongation increases, whereas tensile strength and elastic modulus decreases. TS was lower than soy and higher than whey. Elongation was higher than both. The edible films were strong and elastic, with good physical integrity. The films were shown to be flexible enough to be handled, except for the 80/20 (PPC/glycerol), which produced brittle and fragile films [80]. On comparing the results of Perez et al., a higher PPI/GL ratio gives less transparent films, higher young’s modulus, and maximum tensile strength, but lower strain at break. Bioplastics present higher Young’s moduli and maximum tensile strength than those obtained by soy protein bioplastics, but lower strains at break values [82].

Soy exhibited a greater range of viscoelastic properties based on plasticizers. They have high viscoelasticity and poor tensile and antimicrobial properties compared to whey and albumin plasticized with glycerol. For example, Han et al., demonstrated that the incorporation of CMC to SPI films gave higher tensile strength and lower elongation. CT addition resulted in no significant differences in physical and mechanical properties [98]. Additionally, Mercedes Jiménez-Rosado et al., found that the highest tensile stress was observed at higher temperatures and with the addition of nanoparticles at the same temperature with 1.0 wt.% and 2.0 wt.%, though 4.5 wt.% decreased it. Contrarily, Young’s modulus decreased with an increase in temperature and addition of nanoparticles. Zinc oxide-containing bioplastics showed better properties than those with zinc sulphate. It was reported that all of the formulated bioplastics were strong enough for suitable transport, storage, and distribution of the product [99]. In a similar study, Gamero et al., showed that no effects of lignocellulosic fibers up to 1 wt.% were seen on tensile, elongation, and Young’s modulus, but 5 wt.% had a remarkable increase of 160%, 115%, and 150%, respectively [83]. Research performed by Aguilar et al. (2020) supported that adequate selection of a plasticizer is needed to attain the desired bioplastic. They reported that EG and DEG were difficult to work with due to their volatility issues and major ageing effects during storage. TEG and GLY remained in bioplastics after the tested storage period. TEG was proven to be promising for water absorption applications but produced opaque and brittle bioplastics. GLY provided higher transparency and deformability [100]. Fernández et al., found that a greater content of sodium bicarbonate with SPI/GL blends is easily deformed and exhibits low Young’s modulus due to its large inherent pores [101].

Correspondingly, Liu et al., when studying peanuts, found that heat curing at 70 °C, ultraviolet irradiation for 24 h, ultrasound for 10 min in a water-bath, and formaldehyde and glutaraldehyde addition caused a significant increase in the tensile strength of the films [88]. Patel et al., evaluated that ground nut protein has higher tensile strength than LDPE. As the concentration of protein increases, stiffness and young’s modulus increase, while TS and elongation decrease. It was found that 85% of the film degraded in 60 days by B. megaterium in soil [102]. Studies performed by Sun et al., found that peanut protein isolate (PPI) to pea starch increased elongation by three times its value but decreased TS by half the value. PPI was shown to improve the film’s flexibility and opacity and decrease the thickness of the film. The incorporation of 40% PPI into pea starch film is the best option for edible films with smooth and compact structures [103].

Additional studies by Jang et al., reported the impact on rapeseed blend films with the inclusion of grape seed extract that decreased tensile strength, and increased thickness and elongation [104]. Johansson [58] carried out experiments on three cake sheet materials, namely, rapeseed, carinata, and crambe, which were brittle. The results showed that pre-heating slightly increased %E and lowered young’s modulus. A reverse trend was observed on the addition of siccative. Galus et al., found that an increase in rapeseed oil in whey films increased TS, %E, Young’s modulus, opacity, and soluble matter content and decreased mechanical resistance [105]. The RP films with *Gelidium corneum* or gelatin improved the physical properties. TS increased with sorbitol irrespective of sucrose content, increasing GC with 2% RP and decreasing with the addition of an emulsifier. The optimal RP film was found by Jang et al., to be 2% sorbitol/0.5% sucrose as a plasticizer and 1.5% polysorbate 20 as an emulsifier. The film containing 3% RP/4% gelatin had the most desirable mechanical film property, which can be used for food packages. The mechanical properties of rapeseed protein hydrolysate–chitosan were found to be better than rapeseed protein isolate–chitosan films [106].

Works on wheat gluten by Jimenez Rosado et al., showed that alkaline pH favored the highest tensile and elongation values of extruded WG/GL/W samples. Primary works conducted by Gennadious et al., found that reducing agents like sodium sulfite yielded stronger films [107]. Sun et al., worked on thermo-molded wheat gluten with plasticizers. The reports suggested that the addition of a lipid like beeswax decreased tensile strength and Young’s modulus; shrimp shell powder composites increased the TS by twice as much, and decreased the elongation at break. However, calcinated shrimp composites were more effective in improving the above properties due to their high mineral content [85]. Similarly, Thammahiwes et al.’s work also found that the addition of shrimp shell powder and calcinated SSP increased the TS and decreased the elongation at break % compared to native WG films. CSSP delays the degradation process of WG [62].

Lamination with a heat sealable corn zein layer on soy films had higher tensile strength (two-fold) and lower elongation compared to soy protein isolate films and commercial packages. The lower E was due to the type and amount of plasticizer used, but it was not brittle and had acceptable film pouch fabrication, according to Cho et al. [108]. Ikeguchi et al., generated films with different plasticizers that had homogeneous visual properties, good ductility, and uniform thickness [109]. The inclusion of waxes like carnauba and sorghum in zein edible films and coatings by Weller et al., had no significance on tensile strength, but showed a greater E than single-layer film [110]. Additional investigations by Tapia et al., found that the optimal composition of amaranth film was with a pH of 10.5–11.5 and a glass transition temperature of 76–85 °C, providing good flexibility and moderate opacity. Amaranth flour films exhibited greater strain, good barrier properties, and moderate opacity, but low tensile stress [111].

All biodegradable films produced from sunflower protein isolates with different concentrations of phenolic compounds had nearly the same tensile, elongation, and Young’s modulus values to other protein films, though the color of the package was a limitation [112]. Ayhllon et al., studied the effects of five dissolving bases and five plasticizers on the films produced from the isolate of sunflower proteins (ISFP). The use of a strong base had higher tensile and elongation properties. Among plasticizers, 1, 3-propanediol and glycerol gave the highest TS and E, respectively. With nearly the same glycerol content, ISFP films were more elastic and resistant than soy protein isolate films. The variation of the elongation at break increased with the protein content in the film [113].

The use of reducing or pro-cross-linking agent favors the final product of rice with different physio-chemical properties [91]. Recent studies carried out by Alonso et al., illustrated that those mechanical properties (tensile strength, strain at break, young’s modulus) were enhanced with high processing temperatures, but were lower than synthetic polymers like LDPE. Previous studies show similar Young’s modulus and slightly higher tensile strength for crayfish, albumen, and rice husk materials [87]. In a different study, Cheng et al., evaluated the polyblends of cottonseed protein and polycaprolactone (PCL) plasticized by cottonseed oil. The analysis showed an increased young’s modulus, but decreased tensile and elongation, of PCL blends with water and washed cottonseed meal. Both cottonseed oil and coconut oil as plasticizers showed similar tensile strength and elongation values, except that the young’s modulus was higher in cottonseed oil. Nonetheless, both plasticizers performed better than PEG-400 [49].

### 4.4. Comparison of Different Barrier Properties

**Figure 3:**
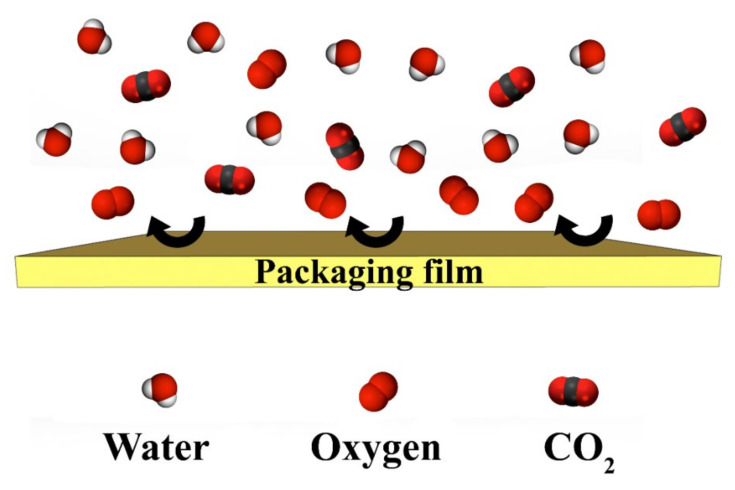
Schematic of barrier properties in packaging films against water, oxygen, and carbon dioxide molecules.

The barrier requirement depends on the end-use applications. Generally, biodegradable polymers possess some drawbacks with respect to conventional packages in terms of moisture barrier property. As it is described in Figure 3, we can see a general schematic barrier property of packaging film against oxygen, water vapor, and carbon dioxide, which are three important gases in the packaging related studies. The addition of plasticizers reduces the chain polymer interactions, improving the barrier properties, thus improving the ductility and flexibility. Likewise, multilayer films, changing conditions, and reinforcements improve the barrier properties. Lower oxygen and moisture barriers are imperative in ideal food packaging to preserve dry, moist, or textured products.

Works carried out by Sharma et al., on edible sesame films show they have low water vapor permeability (WVP) compared to mung bean, lentil, pea, soy, peanut, and fava bean protein [95]. WVP significantly increased with increasing protein concentrations from 3 to 9% due to the hydrophilic character of the film, while it decreased when the pH was increased from 9 to 12. Temperature and glycerol concentration were the other factors affecting WVP. In a similar kind of study, Bourtoom et al., demonstrated that WVP is affected by the pH, temperature, and heating time of the mung bean film solutions. It is also evidenced that the hydrophilic behavior of proteins affects the films. WVP was lowest at a higher pH (9.0–10.0) and increased when the pH was 8.0. An increased heating temperature of the film solutions resulted in a lower WVP. The highest WVP was at the lowest heating time of the film solution [96]. The WVP values of lentil edible films were higher than plastic films, wheat gluten, and corn zein. Bamdad et al., concluded that future works on cross-linking treatments would be helpful to overcome this shortcoming [97].

According to Choi and Han et al., the WVP values of pea were independent of an increased plasticizer content from 20 to 40% but varied on 50/50 protein/plasticizer content. Thus, increasing the plasticizer reduces the barrier properties with an impact on relative humidity explaining good moisture barrier properties [80]. Acquah et al., disclosed that yellow pea isolate (YPI) and yellow pea concentrate (YPC) have promising physio-mechanical properties compared to whey protein isolate (WPI) films. The presence of other non-protein constituents in the matrix of the YPC protein film also made it brittle compared to the YPI, YPI + WPI, and WPI protein films. The moisture content increased 42-fold for WPI, 27-fold for YPI and YPI + WPI, and 16-fold for YPC [114].

Han et al.’s work showed that soy protein isolate (SPI) and SPI/CMC had higher WVP than catechin (CT)-added films, and this effect is comparable to the fatty acids or waxes present in hydrophobic materials. On the other hand, SPI and SPI–CT showed higher oxygen permeability (OP) than CMC-added films [98]. A comprehensive analysis by Liu et al., of peanuts revealed that WVP and OP decreased after heat denaturation and aldehyde treatment. OP also decreased with ultraviolet irradiation [88]. WVP and WVTR decreased significantly with the addition of 40% peanut protein isolate to pea starch. Investigative works conducted by Jang et al., demonstrated that WVP increased with increasing grape seed extract in rapeseed–gelatin blend films. The reason for the increase was suggested to be due to the loss of intermolecular interactions and changes in the average pore size of the film due to the addition of GSE [104].

Further analysis by Jimenez et al., demonstrated that an increase in pH and the presence of additives in wheat gluten improved the water uptake capacity and increased both mechanical strength and water barrier properties. In another study, Gennadios et al., showed that low oxygen permeability was experienced with different soaking treatments (15% lactic acid, calcium chloride, and 7.5 buffer) and partial substitution of wheat gluten (hydrolyzed keratin, the addition of a non-polar hydrophobic substance (mineral oil)). The OP of all the films prepared was low, and film containing keratin had about 80% less OP than that of the control film. The water vapor permeability was decreased by mineral oil (25%), hydrolyzed keratin (23%), and others, except for sodium sulfite [84,107]. Wheat gluten has satisfactory oxygen barrier properties, but not WVP. Hence, it could be used as packaging for lipid oxidation foods, or as a replacement for multilayer laminates like polyamide and ethylene vinyl alcohol. The addition of mineral oil/nonpolar hydrophobic substance decreased WVP by 25%; reducing agent (sodium sulfite) yielded stronger films, but there was no improvement on WVP and OP; soaking films at 7.5 pH increased both strength and water vapor barrier; and tanning agent (lactic acid) had no improvement on the barrier, and only applied to non-edible packs. Wheat gluten-coated papers for bio-based food packaging developed by Guillaume et al., led to a significant improvement of the gas barrier properties of paper by reducing gas permeability by around 30-fold for surface-treated paper, and at least 100-fold for untreated paper [115]. Ciapponi et al., showed that the nature of plasticizers with a longer paraffinic chain decreased the permeability of films against water [116].

Experimentation by Cho et al., on the oxygen permeability of the zein bilayer film showed that it was lower than the commercial packaging, and increased the shelf life by preventing oxidative rancidity for longer than nylon–metallocene linear density polyethylene packaging [108]. In a similar type of work, Ikeguchi et al., showed that zein with oleic acid and olive oil had lower WVP due to a more structured and homogeneous matrix [109]. The inclusion of a lipid layer on dried zein films improved the water barrier properties. Weller et al., found that the coating method of application yielded better results than the emulsion technique. The presence of additives led to a limited swell ability in terms of retaining water, and less deformable material. Besides this, the bilayer film was heated at 120–130 °C with a sealer strength greater than 300 N/m [110]. Amaranth films had better barrier properties compared to other protein and polysaccharide films, according to the work accomplished by Tapia-Blacido et al., The better barrier properties was due to the presence of native lipids and proteins despite the waxy starch [111]. In another investigation, Salgado et al., worked on sunflower protein films naturally activated with antioxidant compounds. They revealed that the WVP of all sunflower protein films had a similar value to that of soy. This is useful for preserving oxidation-sensitive products, as well as in mulching with herbicide if the properties are adequate [112].

### 4.5. Film Solubility and Water Uptake Capacity

Protein concentration, pH, temperature, and choice of plasticizers determine the solubility and the water uptake capacity, which is constitutional according to the applications. Close examination by Bourtoom et al., on mung beans showed that both film and protein solubility decreases significantly when the pH of the film solution rises above 9.0 and the heating temperature is increased from 60 to 80 °C [96]. Another examination by Bamdad et al., determined that the solubility of lentil protein concentrate films was lower than peanut films at pH 9–9.5, denoting its positive stability. The soluble matter was comparable to pea protein concentrate and higher than whey protein isolate films. Conclusively, LPC can be used as hot water-soluble pouches due to high solubility and can replace soy and whey protein in food coatings or edible films [97].

Perez et al., investigated the solubility and total soluble matter of pea and found it to be higher than soy and whey protein [72]. A study by Carvajel et al., showed that higher pea protein isolate and glycerol ratios have a faster water absorption rate, with maximum absorption values being reached within the first 2 h of immersion, with no further increase seen after 24 h, demonstrating their potential for the development of absorbent materials. Water uptake capacity is independent of mixing speed and time. It is 125% higher than potato and rice bioplastics (25% and 30%, respectively). A loss of soluble matter up to 50% due to glycerol is observed because of its hydrophilic character [82].

Recent studies by Jimenez et al., showed that an increase in both temperature and the percentage of zinc oxide nanoparticles reduced the water uptake capacity due to lower free volume and greater cross-linking of systems, but their superabsorbent quality was not compromised [99]. Jimenez et al., also demonstrated that an increase in mold temperature makes the bioplastic lose its superabsorbent capacity (<1000%) and DHT, and US also decreased the water uptake capacity, but not its functionality [48]. In a similar type of study, Aguilar et al., experimented on different polyols as plasticizers in soy-based bioplastics. They found that all of the plasticizers are lost after water immersion, which might be due to evaporation at the drying stage before immersion or through solubilization when immersed [100]. Fernandez et al., developed superabsorbent matrixes through the addition of salts; higher water uptake is seen after replacing sodium bicarbonate for sodium carbonate, which might be due to the higher alkalinization of the material as well as the high hydrophilicity of the carbonate. Water uptake capacity increased after freeze-drying the samples and second immersion but fell short of the original value after a third immersion. Moreover, 1–2.5 wt.% sodium bicarbonate or carbonate with soy protein isolate exceeds the limit of superabsorbent materials [101]. In another study, Gamero et al., revealed that water uptake capacity decreased up to 185% on the addition of 5 wt.% of LCF due to structural reinforcement and could be linked to a lack of flexibility that prevents its swelling capacity [83]. Han et al., revealed that soy protein control films had the lowest water solubility, with CMC having more water swelling, whereas catechin was insoluble in water and had no influence on the water solubility of the films [98].

According to a study carried out by Patel et al., different types of film treatment impact water solubility. The heat curing method gave stronger and high water-resistant peanut films with decreased water solubility, whereas ultrasound treatment increased water solubility [102]. During their study on rapeseed, Johansson [58] commented that water absorption decreases slightly with milling time for rapeseed cake and CR, but increases for CA, and increases slightly for RC with a plasticizer compared to CR sheets. Additional studies by Galus et al., showed that rapeseed oil enhanced hydrophobicity and reduced the moisture content and film solubility characteristics of whey films [105]. Delgado et al., worked on the development of bioplastic materials from the rapeseed oil industry by-products and suggested that rapeseed protein isolate needed to be properly hydrolyzed and the degree of hydrolysis needed to be controlled to improve the solubility of the protein in acidic pH. Chitosan is a hydrophilic polymer and has poor water resistance. An increase in temperature increases its viscoelastic properties and decreases WUC. The WUC decreased with an increase in temperature for all protein systems (RF, M-RM, MS-RM) [89].

More detailed discussion by Rocca-Smith et al., inspected wheat gluten and concluded that all surface analyses of wheat gluten displayed a hydrophilic nature. The incorporation of a lipid phase was able to reduce the water sorption, water affinity, and water transfer in wheat gluten films. All films, including lipids, were characterized by a physical change from a glass-like behavior to a rubber-like behavior when an Aw of 0.43 was exceeded. The water wettability of WG-coated papers was reduced compared to uncoated papers, but to a higher extent for WG–TP (30-fold less) than for WG–UTP (6-fold less). Guillaume et al.’s work on WG–UTP showed that it appeared more wettable by water than WG–TP (20-fold more) because of both a higher water absorption rate and spread ability [115]. According to Alonso et al., the absence of water in the formulation led to higher porosity and WUC, but the plasticizer was not retained in the structure, resulting in more significant matter losses. Blends prepared using glycerol and water exhibited lower viscoelastic properties and better process ability than those only containing glycerol. Furthermore, the sugar-containing blends presented lower solid-like behavior due to their plasticizer effect [117].

Cho et al., worked on edible oxygen barrier corn bilayer film pouches. Only 50% of the film dissolved at 90 °C for 150 s, though the dissolution speed of the bilayer increased as the water temperature rose [108]. Zein films with oleic acid had low wettability and low solubility compared to buriti, olive, and macadamia oil. The choice of plasticizers is a major factor affecting the composition, structure, and morphology of materials, and all three showed homogeneous visual aspects, uniform thickness, and good ductility [109]. In addition to this, Salgado et al., worked on sunflower protein films. The lower the phenolic content in the sunflower isolates, the higher the surface hydrophobicity. All of the values were higher than soy. The solubility of the sunflower films was similar in all buffers, but Sun Iw and Sun Ir were more soluble in water than Sun I [112]. Ayhllon et al., validated the optimum solubilization of SFPI at pH 12. Solubilization with high protein increased with an increase in stirring time [113].

### 4.6. Thermal Properties

Glass transition, crystallization temperature, melting temperature, and specific heat capability are the major fundamental thermal properties considered reliant upon production and application processes, storage, and distribution. It is influential on deciding how the material reacts to the excess or low heat fluctuations.

Sesame protein isolate had better thermal properties compared to other plant proteins like peanut, soy, lentil, and faba bean, as illustrated by Sharma et al., Film with 9% protein concentration, 12 pH, 90 °C, and 10% glycerol displayed better properties [95]. Studies to develop protein blend films or bioplastics from yellow pea proteins showed the absence of multiple Tg due to the reaction of the plasticizers with the pea protein. However, YPI + WPI films had the lowest glass transition not indicative of a synergistic effect on the properties of protein, and there was no difference in the Tg values for protein films formulated with WPI, YPI, and YPC (*p* > 0.05). Whey protein isolate films had better thermal properties, whereas yellow pea isolate, and yellow pea concentrate films showed better protection in terms of limiting light transmission. However, the blend of the proteins did not have a synergistic effect [114]. The thermal degradation of soy was similar to whey and lower compared to albumin, as shown by Jones et al. [19]. There was a reduction in Tg from 67 to 55 °C on increasing sodium bicarbonate from 0 to 10 wt.% [101].

The good interaction and strong adhesion between the fish scale powder and wheat gluten matrix studied by Thammahiwes et al., affected the degradation rate of the wheat gluten bioplastic. This could be used as a control in the agricultural field. Further, it caused weight loss due to 120 h of accelerated weathering [118]. An increase in the plasticizer content decreased the denaturation temperature. A temperature above the denaturation temperature of SFPI, 160 °C die, 20 rpm screw speed, highest glycerol content (70 parts), and medium water content (20 parts) gave most plastic-like and homogeneous films [86].

### 4.7. Other Properties

The addition of additives into a formulary (plastic compound) enhances the functional property, performance, and aging properties of the polymer. The most commonly used additives are functional additives like plasticizers, fillers, colorants, and reinforcements.

Plasticizers like antioxidants, thermal stabilizers, antimicrobials, and curing agents aid in the improvement of the flexibility, durability, and stretchability of polymeric films.

Perez et al., focused on the addition of an antimicrobial (nisin) to the pea protein mixture and found that the films had better antimicrobial properties against Gram-positive microbes (*S. aureus*), giving rise to the microbial preservation of bioplastics. Additionally, an increase in nisin concentration increases young’s modulus and decreases elongation at break; however, there is no change in tensile stress, giving more rigid and less deformable plastics. These tensile properties represent 65, 25, and 35% of the values of strain at break, Young’s modulus, and maximum stress, respectively, for ASTM normalized low-density polyethylene (LDPE) [119].

A considerable impact of additives on soy was seen by Jones et al., when plasticizing albumin and whey with glycerol, which had better antimicrobial properties than the normal soy protein bioplastic [19]. Tulamandi et al., also mentioned in their study that the addition of gelatin to papaya puree gave a glossy finish, high transparency, and tensile strength, but decreased E, Tg, Tm, and ΔH. Defatted soy protein in the papaya/gelatin films improved the elongation, water permeability, and water contact angle values significantly and decreased the water solubility of the edible films [43]. Han et al., worked on a soy protein isolate that had the lowest DPPH radical scavenging activity compared to other films, and its activity was enhanced by CMC. Catechin addition to SPI and SPI/CMC films enhanced the synergistic free radical scavenging effect rising as a potent antioxidant package but had no significant differences in physical and mechanical properties. It was summarized that bioplastic films containing antioxidant agents are effective and safe for food packages [98]. Recent studies by Jimenez et al., showed that the controlled release of nanoparticles and their incorporation, except for 1.0 wt.%, had faster degradation, decomposing into primary elements like nitrogen, serving as supplementary fertilizer [99]. Likewise, the experiment conducted by Gamero et al., on the reinforcement of a small percentage (0.1–1.0) of lignocellulosic fibers had no effect; 5.0 wt.% LCF had worse absorbing properties and higher mechanical properties than the control but were neither homogeneous nor injectable. A 130 °C mold temperature had better reinforcements [83]. Aguilar et al., worked on the different effects of polyols as plasticizers and concluded that bioplastic with tri ethylene glycol was more ductile, opaque, and had higher water uptake capacity. Those with ethylene glycol as plasticizers gave rise to ductile, translucent, and less water-absorbing bioplastics. Both were complex to work with and had major aging effects [100]. TEG or glycerol had promising results for water absorption applications and transparency with greater deformability. Future studies are concentrated on different protein/plasticizer ratios and low processing conditions.

In terms of the effect of additives on rapeseed, Jang et al., tried packaging ‘Maehyang’ strawberries with rapeseed gelatin/1.0% grape seed extract, which decreased the populations of total aerobic bacteria, yeast, and molds in the product after 14 days of storage compared to that of the control. There was a higher sensory score of RG-GSE-packed products compared to all treatments for increasing storage. Additionally, it helped to maintain the freshness and firmness of the product and is recommended for packaging [104]. Changes made by Galus et al., in whey protein-based edible films with rapeseed oil did not affect the appearance of the product in use. It reduced the moisture sensitivity, which positively protects high moisture food products [105]. Works by Jang et al., on the RPH-CH composite films found they could be used in food packaging as they had a stronger antimicrobial activity than CH film against Staphylococcus aureus and Bacillus subtilis compared to Escherichia coli [120].

Several modifications were made by Gennadious et al., on wheat gluten, for instance, films with hydrolyzed keratin had low oxygen and water vapor permeability; films containing mineral oil and soaked in calcium chloride and lactic acid had lower water vapor permeability and higher tensile strength and elongation compared to other films. Treating with a tanning agent (lactic acid) had a plasticizing effect but did not improve the barrier properties [107]. The incorporation of a lipid phase like beeswax reduced the water sorption, water affinity, surface hydrophilicity, water interaction, and transfer. However, Rocca Smith et al., did not recommend only the use of a lipid source, as it would compromise the mechanical properties, making it brittle and fragile [121]. Tan δ max peak height and storage modulus decreased with the addition of wheat gluten bioplastics with fish scale compared to wheat gluten native films by 2.5% and 7.5% wt.%, respectively [118]. The latest study by Alonso-Gonzalez et al., on the incorporation of sugars as plasticizers or fillers increased the water uptake capacity due to higher porosity and had solid-like behavior [117]. Ciapponi et al., studied the presence of microbial biomass (freeze-dried Spirulina platensis) as a filler. They substantiated from their experiment that microbial biomass had no significant change in barrier properties, decreased elongation at break, but improved thermal stability up to 120 °C increased tensile strength and elastic modulus. An increasing amount of biomass decreased the water contact angle, which might be due to its hydrophilic nature [116].

## 5. Conclusions

Worldwide, there is variation in the production and availability of protein sources. We discussed the value presented by plant proteins derived from agri-food industrial biowastes or co-products in the present review. Even though bioplastics promise to be an excellent alternative, there are certain shortcomings in their use for the replacement of conventional plastics. The vast majority of current industrial bioplastic applications rely on cellulose and starch, and neglect protein-rich biowastes. The current assessment of peer-reviewed studies does mention some of their disadvantages and recommends ways to overcome them. We have indexed crude protein content in oilseed cakes and meals, cereals and legumes, vegetable wastes, fruit wastes, and cover crops, along with the top global producers. We also described and compared the different production techniques (casting, extrusion, and molding) as well as the composition of the bioplastics using the best protein sources, such as sesame, mung, lentil, pea, soy, peanut, rapeseed, wheat, corn, amaranth, sunflower, rice, sorghum, and cottonseed. Therefore, bioplastic production is being thoroughly researched by scientists in order to identify the most effective methods for practical applications. The biopolymer industry is well positioned to lead the way towards a circular economy by demonstrating environmental responsibility, biodegradability, feasibility, and reliability.

## Figures and Tables

**Table 1:** Rich protein sources and their global producers. Rich sources of crude protein composition (percent/dry matter basis).

Protein	Source	Protein Percent (%/DM)	Top Global Producers	References
Oilseed cakes/meals	Canola/Rapeseed	33.9	Canada, China, Europe, Russia, Asia (India, China, Indonesia)	[59,60,61,62]
	Coconut	25.2	Malaysia, Indonesia	
	Cottonseed	40.3	Southern US, Brazil, Asia	
	Groundnut	49.5	Asia (India, China)	
	Mustard	39.5	India	
	Olive	6.3	Mediterranean countries of Spain, Italy, Greece, Tunisia	
	Palm kernel	18.6	Malaysia, Indonesia, China	
	Sesame	35.6	Asia (Burma, India, China), Africa, South America	
	Soybean	47.5	North and South America (USA, Brazil and Argentina), Asia	
	Sunflower	34.1	Russia, Ukraine, Argentina, USA, China, India and Turkey	

**Table 2:** Protein sources with the total protein content, their distribution and top global producers.

Protein	Source	Total Protein Content (%)	Protein in Other Parts (%)	Top Global Producers	References
Cereals and Legumes	Barley (*Hordeum vulgare* L.)	12.5	Hay (10–15), Grain (11–15)	Fourth most widely grown in the world	[59,60,61,62]
	Corn (*Zea mays*)	40–50	Kernel (7–12), Silage (8–11)	Latin America, Africa, Asia	
	Wheat (*Triticum*)	7–22	Flour (9–13), Bran crude (15.5)	Asia (China, India), Russia, United States	
	Rice (*Oryza sativa*)	7	Bran (13)	Asia (China, India, Bangladesh)	
	Soybean (*Glycine max*)	42	Hay (17), Defatted flour (50–59)	Brazil, United States, India	
	Mung bean (*Vigna radiata*)	16–23		Asia (India, China, Myanmar)	
	Sunflower (*Helianthus*)	20–28	Silage (11–12), Seeds (16.7), Hulls (6.2)	Russia, Ukraine, Argentina. EU-27 countries, China, USA	
	Peanut (*Arachis hypogaea*)	38.11		Asia (India, China)	
	Sorghum (*Sorghum bicolor*)	22	Hay (7), Stover (5)	Asia and Africa	
	Mustard (Vigna *radiata*)	24–35	Hay (10)	India	
Vegetable and fruit wastes	Bottle guard (*Spinacia oleracea*)		Pulp (24.3)	Asia (India, Sri Lanka, Indonesia, Malaysia), South Africa	
	Citrus (*Citrus limetta*)		Pulp (without peels) (10.5)	Brazil, China, Mexico	
	Guava (*Psidium guajava*)		Seeds (7.6), Guava seed protein isolate (96.7)	India, China, Thailand	
	Peas (*Pisum sativum*)		Pea pods (19.8), Pea vine (11.8), Pea straw (5–10)	China, India, USA, France, Egypt	
	Snow peas (*Pisum sativum saccharatum*)		Culled (23.2)	Russia, China, Canada, Europe and fourth in worldwide	
	Sugar beet (*Beta vulgaris*)		Leaves (21.9), Pulp (10.0)	Russia, France, USA, Germany	
	Tomato (*Solanum lycopersicum*)		Pomace (19–22), Culled tomatoes (14–20), Seeds (24.5)	China, India, USA, Turkey, Egypt	

**Table 3:** Protein-rich cover crops in Canada.

Protein Source	Protein Percent in Grain (%)	Crude Protein in Other Parts (%)	References
Amaranth (*Amaranthus* sp.)	14		[63]
Beet (*Beta vulgaris*)	8.9	Tops 12–15, root 7–10	
Berseem clover (*Trifolium alexandrinum* L.)	27–29		
Canola (*Brassica napus*)	21	Hay (16), silage (12), pasture (17), hull (15.2)	
Cereal rye (*Secale cereale* L.)	14	Straw (4)	
Chickpea (*Cicer arietinum* L.)	22	Straw (6)	
Chicory (*Cichorium intybus* L.)	10–32		
Cowpea (*Vigna unguiculata* L.)	19–24		
Field pea (*Pisum satuvum arvense* L.)	24	Silage (15) Hay (14)	
Kale (*Brassica oleracea*)	30		
Lentil (*Lens culinaris Medik*)	28	Hay (14) Silage (15)	
Lupin (*Lupinus* L.)		Silage (15)	
Medic (*Medicago* sp.)	Black medic 19–21		
Mustard (*Brassica* sp. L.)	24–35	Hay (10)	
Oats (*Avena sativa* L.)	13–18	Kernel (40–60), hay (9–15)	
Radish (*Raphanus sativus*)	26–30		
Safflower (*Carthamus tinctorius* L.)	18	Hay (10–13)	
Spinach (*Spinacia oleracea* L.)	20		
Sweetclover (*Melilotussp*. L.)		Hay (11–18)	
Triticale (*Triticale hexaploide Lart*)	17	Hay (9–16)	
Turnip (*Brassica rapa*)		Tops (16), root (12–14)	
Vetch (*Viciasp*.)	13–20		
Wheat (*Triticum aestivum* L.)	12–16	Straw (4–10)	
White clover (*Trifolium repens* L.)	24–30		

## Data Availability

Not applicable.

## Supplementary Materials

The following supporting information can be downloaded at: https://www.mdpi.com/article/10.3390/polym14051065/s1, Table S1: Protein sources with their type, method of formation, and composition of bioplastics; Table S2: Comparison of various properties of bioplastics (data in connection with Table S1).
