# Supplementary material for: Comparison of Protein Content, Availability, and Different Properties of Plant Protein Sources with Their Application in Packaging
Source: Polymers (Basel). 2022 Mar 7;14(5):1065. doi: 10.3390/polym14051065 (PMC8915110; doi:10.3390/polym14051065)
Supplement: Supplementary file 1 [file polymers-14-01065-s001.zip › polymers-1599164-supplementary-done.pdf]

polymers

Supplementary Materials

Comparison of Protein Content, Availability, and Different Properties of Plant Protein Sources with Their Application in Packaging

Anupriya Senthilkumaran [1], Amin Babaei-Ghazvini [1], Michael T. Nickerson [2], Bishnu Acharya [1,*]

[1] Department of Chemical and Biological Engineering, University of Saskatchewan, Saskatoon, SK S7N 5A9, Canada; jwy763@mail.usask.ca (A.S.); amin.babaei@usask.ca (A.B.-G.)
[2] Department of Food and Bioproduct Sciences, University of Saskatchewan, Saskatoon, SK S7N 5A8, Canada; mtn620@usask.ca
* Correspondence: bishnu.acharya@usask.ca

Table S1: Protein sources with its type, method of formation and composition of bioplastics

S.No	Source	Type	Method of formation: Castin	Method of formation: Extrusi	Method of formation: Molding	Composition/parameters	References
1	Sesame	Edible film	✓			Sesame Protein Isolate (SPI) from defatted sesame meal by alkali method. SPI (3, 6, 9%) + distilled water +plasticizer [glycerol{GLY} (10, 30, 50%w/w on protein)], pH 9-12, heated at 70-90°C, dried at 60°C and 50% relative humidity(RH) for 8 h	[88]
2	Mung bean	Edible film	✓			Protein from mung bean flour by alkaline extraction and acid precipitation. Freeze-dried proteins+ distilled water(3g/100ml) + pH 8 -10+ plasticizer [sorbitol 2:1] Homogenized at 10,000rpm for 2minutes, heated at 60,70,80° C, cooled to ambient temp and stored at 60% RH	[82]
3	Lentil protein	Edible film	✓			Lentil protein concentrate (LPC) from lentil seeds LPC(5g/100mlwater), plasticizer [GLY 50%(w/w) of LPC)], pH 11, heated at 70°C for 20mins, dried at 60°C for 7hr	[83]
4a	Pea	Edible film	✓			Denatured pea protein concentrate (PPC) from yellow field peas acquired as a liquid. Aqueous solutions of 10% (w/w) PPC+ Plasticizer [GLY/ g PPC] at a ratio from 80/20 to 50/50 of PPC/Glycerol. Solution heat denatured at 90°C for 25mins and cooled to room temperature	[66]

b		Bio-plastic		✓	Pea protein isolate directly acquired Protein/plasticizer (1.5,1.6,1.7) [GLY,GLY+ nisin (0,2,4%)] Mixing (two-blade counter-rotating batch mixer) under adiabatic conditions at 25°C,50 rpm for 5 minutes. Injection molding (MiniJet Piston Injection Molding System II) under50MPa, pre-injection at 60°C,100s and packing stage at 50 MPa and 130°C	[105]
c		Bio-plastic		✓	Pea protein isolate directly acquired Blending of PPI/GLY ratios (80/20; 70/30; 60/40 and 50/50) using a two-blade counter-rotating batch mixer (25°C and 50 rpm for 60minutes) to a dough-like blend & injection molding [MiniJet Piston Injection Molding System (ThermoHaake)] with pre injection cylinder at 50°C for 100s,0.1MPa, injection at 50°C,0.1-50MPa and <1s. Packing stage with 130°C,50MPa and 200s	[58]
d		Bio-plastic		✓	Pea protein isolate (PPI) is directly acquired. PPI/GLY (60:40) mixed in two-blade counter-rotating batch mixer with speeds (10, 30, 50, 70 or 90 rpm) and time (from 1 to 180 min depending on the system) at 25°C, generally 10min is set for all RPMs, 1 and 65 min additionally for 30 rpm. The injection cylinder was 130°C.Mold at 50°C, residence time for20s at 500 bar pressure, post-injection time at 200sand 200 bar Pr.	[68]
e		Bio-plastic	✓		Yellow pea concentrate (YPC), pea protein isolate (PPI), whey protein isolate (WPI) was directly acquired. WPI, YPI, YPC and YPI + WPI (1:1 v/v) at 4% (w/v) with de ionized water. Solution adjusted to pH 8.0, heated at 75 °C for 30 min at 30 rpm. GLY (50% w/w) added. Films dried at 25°C for 72hr and stored at 50-52% RH	[100]
5a	Soy	Bio-plastic		✓	Soy protein: plasticizers (GLY, water, natural rubber latex) [3:1] Compression molding on bench-top press (Carver Model 3850, Wabash, IN) at 120°C for 5 min followed by 10 min cooling and conditioned at 21.1°C, 65% RH for 24hr	[13]
b		Edible film	✓		Defatted soy protein (DSP), Gelatin(G), Papaya puree (PP) procured directly 1. Control films [8g w/w to 100ml of water with 2g w/w starch], 2. PP/1%G, and 3. PP/2%G, 4. PP/3%G, 5. PP/4DSP, 6. PP/1G/4DSP, 7. PP/2G/4DSP, 8.PP/3G/4DSP.Films dried at 40°C,23%RH for 18hr and stored at 25° C	[37]
c		Bio-plastic	✓		Soy protein isolate (SPI) 1.[SPI(7g/100mlwater) +GLY (3.5%w/v)], 2. [SPI (7g/80ml water) /CMC (0.5g in 20ml water), 3. [0.15% w/v catechin to 1], 4. [0.15% w/v catechin to 2] Films dried and stored at 25°C, 50% RH for 24 h.	[84]
d		Films		✓	Soy protein isolate (SPI) is directly acquired. SPI and GLY (1:1) mixed in two-blade counter-rotating Thermohaake mixer for 10mins,50rpm at below 37°C and torque 5Nm. Injection molding of dough like blend	[42]

					in Thermohaake Minijet piston Injection molding system cylinder at 40°C and mold at 70°C injected for 20s at 500 bar using a post-injection pressure of 200bar for 300s. Strengthening treatment- mold Temp increase (70-130°C), dehydrothermal (4 & 24hr at 50°C), Ultrasound (5 & 45 min at 20kHz)	
e		Bio-plastic		✓	Soy protein isolate (SPI)<br>Mixed SPI,GLY(1:1) & Zinc Oxide nanoparticles (0, 1, 2, 4. 5wt. %) for 30 minutes at 50 rpm under adiabatic conditions, beginning at 20°C. Dough-like blends to Injection molding [MiniJet Piston Molding System II cylinder] at 40°CC, 600 bar for 20 s & mold temp 70°C, 90°C or 110°C for 20 s at 200 bar.Finally thermal treatment at 50°C for 24hr.	[85]
f		Bio-plastic		✓	Soy protein isolate (SPI)<br>SPI, additive- Ligno Cellulosic fibers (0,0.1,1.0,5.0wt.%), GLY mixed in a two-blade counter-rotating mixer Polylab QC at RT 30°C50rpm for 10min under adiabatic conditions. Injection molding with cylinder temperature 40°C, mold temperature 70,90,110,130°C, injection pressure 500 bar for 20 s and holding pressure 200 bar for 300 s	[69]
g		Bio-plastic		✓	Soy protein isolate (SPI)<br>Blending of SPI, 45%wt. plasticizer-polyols (GLY, EG, DEG, TEG) at RT,50rpm for 10mins and Injection molding at 120°C,600-700 bar other parameters varied according to plasticizers	[86]
h		Super-absorbent matric		✓	Soy protein isolate (SPI)<br>Mixed SPI/ GLY (1:1) in a 2-blade counter-rotating batch mixer at RT, 50rpm for 10mins.Injection molding [MiniJet Piston Injection Molding system] with cylinder temp of 40°C, injected at 500bar-20s, compression molded at 200 bars for 300s, mold temperature at 60,70,80,90 and 130°C	[87]
6a		Edible film	✓		Peanut protein isolates (PPI) from defatted peanut flour using alkaline extraction and acid precipitation. PPI/GLY solution at pH 9. Casted, dried at 90°C for 16hr and stored at 65% RH. Properties of film modified by physical & chemical treatments	[74]
b	Peanut	Package film	✓		Groundnut rotein(GNP) from de oiled cake using alkali extraction & acid precipitation. Stock: chitosan [Chi (2%w/v)], Polyvinly Alcohol [(PVA) (5% w/v)]. Films [1.GNP3- GNP 3%, Chi 2%, PVA 5%;2. GNP4- GNP 4%, Chi 2%, PVA 5%;3. GNP5- GNP 5%, Chi 2%, PVA 5%]casted, dried at 65°Cand stored at 23°C,60% RH for 48hr	[88]
c		Peanmat-DIY bioplastic			Peanmat- Compound [Peanut hulls waste (17g), potato flour from skins (30g), whole milk(expired), GLY-5ml] cooked in a fan-assisted oven at 180°C for 13 mins.	[108]
d		Edible films	✓		Peanut protein isolates (PPI) by alkaline extraction and acid precipitation.	[89]

						Films [Pea starch: PPI (1-100:0%, 2-90:10%, 3-80:20%, 4-70:30%, 5-60:40%,6-50:50%, 7-0,100%) +GLY (1.5g)]/100ml distilled water. Solution boiled for 30mins,degassed under vacuum (0.1MPa for 10mins), cooled at RT & dried at 25°C for 24hr	
7a		Package film	✓			Rapeseed protein (RP) powder from rapeseed meal. Film [RP(3g) +G(3g)+sorbitol(2g)+sucrose(0.5g)+1.5%polysorbate 20+Grapeseed extract (0.5,0.7,1.0,1.2,1.5%)]casted and dried at 25°C for 48hr	[90]
b		Tray			✓	Rapeseed cake (RC) is directly acquired. Mixing of preheated RC+15% GLY & compressed to sheets at 100°C, 220bar pressure for 12mins with siccative Manganese (III) acetylacetonate of 0 and 0.1wt. %.10g of sample was Extruded using Haake Rheomex twin extruder at 100°C and screw speed 35rpm.	[109]
c		Edible films	✓			Rapeseed oil directly acquired Denatured whey Protein Isolate (8%) + GLY(50%) + Rapeseed oil (0,1,2,3%) homogenized at 13,500 rpm for 6mins.Casted,dried at 25°C, 50%RH for 24 hr and stored at 54%RH, 25°C	[91]
d		Edible films	✓			Rapeseed protein (RP) from rapeseed oil residue and Gelidium corneum algae (GC, gelatin (RG) directly acquired. 1. RP 4% (w/v)+ sorbitol (1.5,2%)/ sucrose (0.5,1.0,1.5%) + emulsifier polysorbate 20 (0.5,1.0,1.5,2.0 wt.%). 2. RP (2, 3, 4, 5 wt.%), GC (0.7%), 3.RP(2 wt.%),GC(0.5,0.7,1.0,1.3,1.5 wt%) Solution casted and dried at 25°C for 48hr	[106]
e	Rapeseed	Composite films				Rapeseed protein isolates (RPI) prepared from protein meal. Rapeseed protein hydrolysate (RPH) by using alkaline protease. Films of RPI, RPI-Chitosan (CH), RPH [3, 6, 9, 12% hydrolysis]-CH, 12%RPH:CH (2:8, 4:6, 5:5, 6:4 ratio) +GLY was given ultrasonic treatment for 10mins at 65W and dried at 25°C,55% RH	[92]
f		Bioplastics			✓	Rapeseed meal (RM), milled pelletized-RM (M-RM), milled and sieved pelletized-RM (MS-RM) were directly acquired. protein:GLY60:40) mixed in two-blade counter-rotating rheometer at 25°C, 50rpm for 15mins. Injection molding at T(80,100,120°C), injection pressure with 500bar for 20s, holding pressure of 200bar for 300s. T of injection chamber 60°C in all cases.	[75]
g		Edible films	✓			Rapeseed protein isolate(RPI) from defatted rapeseed meal. Coarse solution (4% w/v) fromlyophilized RPI or SRPI [5,10,15% (w/w) of succinic anhydride added to RPI]. Films casted and dried at 25°C,50%RH for 48hrs	[110]
8a		Bioplastic		✓		Wheat gluten directly acquired Mixing of WG 50wt.% +glycerol 18wt.%+ water32wt.% + glyoxal 3wt.%+Xanthan gum 1.5wt.% at	[70]

					pH9 introduced into Rheomex 302p screw extruder with spindle speed 30rpm, die-cutting, compression molded at 130°C, 9MPa for 10min & conditioned for 2 weeks at 53%RH	
b	Wheat gluten	Edible films	✓		Wheat gluten (WG) directly acquired. Films with different soaking treatments. Control-WG (15g) +ethanol (72ml) + glycerol (6g), 1. Mineral oil (3.5g); 2. Sodium sulfite (0.2g); 3. 2g replaced by keratin in 15gWG; 4. Control soaked in 15% w/w lactic acid; 5. Control soaked in 1M Calcium Chloride; 6. Control soaked in buffer solution for 20s with pH 7.5. All dried at 32°C for 5hr	[93]
c		Edible films	✓		Wheat gluten was directly acquired. Emulsified WG with lipid phase (a blend of acetic esters of mono and di-glycerides and beeswax). 1.WG (10g) +plasticizer(glycerol-2.5g) + ethanol and HCL (50g) with ultrasound treatment for 12mins, thermally treated at 70°C for 15mins and dried at 25C for 20hr, 2.EWG films+ lipid phase+ emulsifier-glycerol monostearate melted at 70°C, dried at 25°C for 24hr and 40°C for 1.5hr in oven. Finally, both dried at 53%RH,25°C	[107]
d		Bio-plastics		✓	Wheat gluten is directly acquired.<br>Mixed WG+ glycerol(30wt.%) +fish scale wastes (0, 2.5 ,5, 7.5, 10 weight%) in two-roll mill at RT for 2mins and compressed at 130°C for 10mins with pressure 250kg/cm^3 in molds	[104]
e		Bio-plastics		✓	Wheat gluten (WG) is directly acquired.<br>Mixed WG: glycerol (100:35) in 3-roller mixer at room temperature, cold-pressed at 15MPa for 5mins, thermo-molding at 65,85,105,125°C at 15MPa for 15mins and stored at 52% relative humidity	[71]
f		Bio-plastics		✓	Wheat gluten is directly acquired.<br>Mixed WG (10-wt.%) +glycerol (30 weight% of dry WG), shrimp shell powder (0,2.5,5.0,10wt.% of WG) / calcinated shrimp shell powder (2.5wt.%) in two roll mill at RT for 2minutes & compression molded at 130°C,10minutes, pr 250kg/cm^3	[55]
g		Coating			Wheat gluten (WG) directly acquired<br>WG-coating solution- WG 20g +50ml deionized water + reducing agent (Sodium sulfite 0.08g/100ml) +pH 4 on the surface of the treated and untreated paper using thin-layer chromatography applicator & stored at 25°C for a week	[101]
h		Super-absorbent matrix		✓	Wheat gluten is directly acquired.<br>6 samples with WG, plasticizer-water and glycerol disaccharides -sucrose and trehalose. Mixing at 2-5rpm increased till 200rpm at 25°C for 5 then injection molding in Haake pneumatic piston with cylinder at 50°C & mold T-130°C, injection and holding pressure 500bar for the 20s and post-injection 200s followed by dehydrothermal treatment at 50°C for 24hr	[111]

i		Bio-plastics			✓	Wheat gluten directly acquired<br>Preheating mixture at 80°C for 2mins. Mixing of WG, Glycerol, Octanoic acid, and 1,4-butanediol, micro algal biomass (10,20,30 per hundred parts) and hot pressed in compression molding at 120°C,40 bar for 10mins	[102]
9a	Corn zein	Edible film pouch	✓			O_2 barrier bilayer film pouch for Olive oil condiments.<br>Corn zein and Soy protein isolate (SPI) were directly acquired. Homogenized corn zein layer (2g CZ & 0.6g PEG in 30ml ethanol) was poured to dried SPI film (5gSPI,1.25g glycerol & 1.25g sorbitol in 100ml water) dried at 80-85°C for 30mins	[94]
b		Bio-films	✓			Corn zein directly acquired.<br>Granular zein in 75% ethanol solution to a concentration of 20 % (w/v) +plasticizer [oleic acid or edible oils] (0.7%) + additional plasticizer- glycerol (0.3%), + emulsifier (0.05%). Solution heated at 60-65°C for 10min, maintained at 25°C for 48hr & stored at 58%RH	[95]
c		Edible bilayer film	✓			Double casting,<br>Zeinfilm:Corn zein+ aqueous ethanol+ plasticizer(glycerin+ PEG600) (135,795,3,33g/kg concentration respectively) heated at 60°C, stored at 25°C,50%RH for 24hr.Bilayer films- by coating dried zein films with medium-chain triglyceride (MCT)oil, sorghum wax(SW)/MCT oil or carnauba wax(CW) /MCT oil & conditioned for 2days at 50%RH at 25°C	[96]
10	Amaranth	Bio-films	✓			Amaranth flour by alkaline wet milling<br>4%Amaranth+ plasticizer (glycerol30,35,40g/100g flour):	[97]
11a	Sunflower	Films	✓			Three sunflower protein Isolate from residual pellet SPI (5%w/v) + plasticizer (1.5% w/v glycerol), pH 11dried at 60°C for 5hr and stored at 20°C,58%RH	[98]
b		Films		✓		SFPI by alkaline extraction from oiled cake<br>Mixing of [SFPI 100 parts by weight, plasticizer (glycerol 10-70), Water 10-50], conditioned at 25°C for 12hr. Extrusion-single screw extruder (width-10cm, thickness 0.05 and 1mm, Tdie-85C-160°C, Tsupply tank-50°C, screw-seed 20-200 rpm, dia19mm, L/D-25, compression rate-1.8)	[72]
c		Films			✓	Isolated sunflower protein from sunflower oil by alkali.<br>Mixing of ISFP/ plasticizer-glycerol (2:1) + additives (1 to6%) [aldehydes, plant tannins, fatty acids & fatty alcohols] Thermo molding at 155°C for 2min using a hydraulic press, cooled 5mins & stored at 25°C,60%RH	[76]
d		Bio-films	✓			Isolate of protein (IFSP) from oil cake by alkali extraction.<br>ISFP(2g) in 20ml of water, pH adjusted to 12 with bases [lithium hydroxide (LiOH), sodium hydroxide (NaOH), potassium hydroxide (KOH), aqueous	[99]

					ammonia (30%), and triethylamine (TEA)] + plasticizer [five plasticizers (Glycerol,1,3-propanediol, D-sorbitol, triethylene glycol (TEG), tetra ethylene glycol (TEEG)] stirred at 19000rpm, 1 min & centrifuged 5000rpm,5mins to remove air bubbles, dried at 25°C for 24hr	
12a	Rice	Bio-plastic		✓	Rice protein isolate from rice husks. Mixed RPC+ plasticizer(glycerol) +additives [reducing agent-Sodium bisulphite 0.3 wt. %; cross-linking promoters-glyoxal 3.0 wt.% and L-cysteine 1 wt.% in two-blade mixer at 50rpm,60 min starting at 25°C] & dough-like blend in Injection molding [MiniJet Piston Injection Molding System (ThermoHaake)] at 130°C 300sec,95MPa,	[77]
b		Bio-plastic		✓	Mixed Rice protein + plasticizer (glycerol-33 & 43 wt.%) in Polylab torque-rheometer by two rollers at 50rpm,15mins, compression molding at 100 bar pressure for 10 min at 90,100,120,140,160 & 180°C) & stored at 53%RH	[78]
c		Bio-plastic		✓	Rice bran is directly acquired. Mixing [55% RB and 45% total plasticizer (2:1 water/glycerin)] in 2 blade counter rotating batch mixer at 200 rpm,80°C & injection molding at three T=100,130,150°C with cylinder temperature at 50°C, injection pressure at cavity 500 bar& injection time 15s	[73]
13	sorghum	Films	✓		Kafirin extracted from sorghum gluten with hot ethanol (wet milling) 25g kafirin+105.5ml of 95% ethanol+ plasticizer (6.08g glycerin & 5.48g of PEG). Stored at 25°C & 50% RH	[79]
14a	Cottonseed	Bio-plastics		✓	Glandless cottonseed flour directly procured. Mixture of denatured cottonseed protein and GLY mixed using speed mixer and three roller mills followed by hot press molding at 20 MPa,130°C for 5 minutes	[112]
b		Bio-plastics		✓	Gandless cottonseed flour and cross-linking agents-formaldehyde glyoxal, glutaraldehyde was directly acquired. GLY+ Dried denatured cross-linked protein in high-speed mixer for 5 minutes, ground in triple roller mill and conditioned for 24hr at RT & hot press molded at130°C, 200MPa for 5 minutes	[59]
c		Bio-plastics		✓	Cottonseed protein isolate (CPI) from the defatted seed of glandless cotton by base solubilization and acid precipitation Mixing Polycaprolactone + protein (Washed Cottonseed Meal, CPI, Soy PI) + plasticizer (cottonseed oil, coconut oil, PEG-400) at different ratios, heated to 160°C for 5-10 minutes and hot pressed in molds at 80°C, 10 min & 2.76 MPa.	[43]

Table S2: Comparison of various properties of bioplastics (Data in connection with Table S1)

S. No	OP [cm³·μm/(m²·day·KPa)]	WVP (g.mm/.day. KPa	Tensile strength (MPa)	Elongation at break (%)	Thermal	Young's modulus (MPa)	Solubility (%)	Surface hydrophobicity (UA mL/mg)
**1**		0.010-0.018	0.47-3.03	16.12%	SP: Tg-50°C SPI: Tg-37°C		51.06-87.00	
**2**		11.37-16.91	3.73-6.59	8.83-37.70			Protein:19.26-27.00 Film:37.53-39.43	
**3**		0.0035	4.24±1.26	58.22			low at pH 7.5 and 9	
**4a**		Heat denatured film at ppc/gly (70/30)-0.170, (50/50)-309	Heat denatured film at ppc/gly (50/50)-0.69 ± 0.07, (80/20)-4.93±1.02, native film (70/30)- 4.1±1.73	Heat denatured film at ppc/gly (80/20)-0.8± 0.2 (60/40)-147±13.6 Native film (50/50) - 95.3±20.3		Heat denatured (70/30)-204.86±66.48 Native film-(70/30)-1015.2±187.4	32.2±9.8-37.8±4.5	
**4b**			No change on nisin addition	Decreased with increasing nisin		Increased with increasing nisin		
**4c**			High for 70/30 and 60/40 ratios		PPI: Tg-100°C		PPI:30	Water absorption capacity is high >100%
**4d**			Highest at 30 rpm (1 & 10min)	Have similar values independent of the mixing process		Highest at 30 rpm (1& 10 min)		Water uptake capacity is high (hydrophilic)
**4e**			WPI -1.72 ± 0.61, YPI+WPI-0.43 ± 0.22, YPI-0.65 ± 0.29, YPC-0.18 ± 0.092,	YPI-65.64 ± 29.35, WPI-39.26 ± 13.88, YPI+WPI-35.57 ± 17.79, YPC-9.69±4.84	Tg-YPI+WPI 85.3±4.95°C same range for WPI, YPI and YPC (94.6-95.8°C)	WPI-66.63 ± 23.56, YPI+WPI-28.64 ± 12.81, YPI-6.65 ± 2.97, YPC-0.026 ± 0.013,	YPI-36.5± 5.22; WPI, YPI+WPI, YPC in range of 49.3-51.6 51.6±4.84	Surface hydrophilicity: YPC < YPI < YPI + WPI < WPI

**5a**			Poor with Soy-Gly 7.5 as max		The endothermic peak of soy bioplastic-180-185°C	Soy-gly 300, Soy-NRL 100		
**5b**	Highest in PP control films-8.71, lowest in PP/3G-7.52.	Highest in PP control-8.45 and least in PP/3G/4DSP -5.55	4DSP/3G/PP-6.8, Highest in PP/3G-8.20	Max of 28.11-PP/4DSP, min of 13.15-PP/3G	Tg of PP-90.19, PP/3G-87.75, PP/4DSP-91.22, PP/4DSP/3G-89.11		PP-77.54, PP/3G-88.70, PP/4DSP-72.20, PP/4DSP/3G-82.26	Water contact angle: Highest-PP/3G/4DSP-75º, Lowest-PP /4DSP 25.27 º, PPcontrol-29.34 º.
**5c**	SPI -1.09± 0.03, SPI/CMC-1.08± 0.07, SPI/CT-1.24±0.04, SPI/CMC/CT -1.10 ±0.05 (×10^{-3}cc·mm·m^{-2}·day^{-1}·kPa^{-1})	SPI -19.80±2.48, SPI/CMC -16.32±0.94, SPI/CT-15.93±1.29, SPI/CMC/CT -15.26±0.57	SPI -4.09 ±5.3, SPI/CMC-5.68 ±0.39, SPI/CT-4.33±0.24, SPI/CMC/CT -5.56±0.28	SPI -237.48 ±15.25, SPI/CMC-131.42± 9.64, SPI/CT-247.79± 26.20, SPI/CMC/CT -140.44± 16.65			SPI -27.37±0.73, SPI/CMC -30.54 ±0.49, SPI/CT-28.69 ±0.76, SPI/CMC/CT -29.34 ±0.40	
**5d**			Max: DHT at 24 hr., DHT at 4hr,130°C	Max: DHT at 4hr, 130°C, DHT at 24hr		Max: DHT at 24 hr., DHT at 4hr, 130°C		DHT decreases water uptake capacity
**5e**			Max at highest mold T, 1 and 2wt% ZnO and decreased at 4.5wt.%	The effect is significant at all temperatures and 1wt.% is the highest		Decreases with an increase of Temperature or ZnO content	Solubility had no significant changes to changing conditions	Reduced water uptake capacity with increase in both temperature and ZnO %

**5f**			Max at 5% LCF (160% increase) no differ-ences observed upon 1wt.% LCF	Max with 5%LCF (115%inc) but no change till 1%		Experi-enced 1.5 times higher with 5% LCF no changes until that	No significant differences in soluble matter loss for all systems	Water uptake capacity de-creased from 340 at 0.1 & 1% to 185 at 5% LCF
**5g**			SPI/DEG > SPI/ GLY > SPI/EG > SPI/TEG	Max for SPI/GLY, SPI/DEG, SPI/ EG-ductile, SPI/TEG-frag-ile behavior	Tg for SPI/EG, SPI/GL Y, SPI/DE G and SPI/TEG is <30,75, 80, 105°C respec-tively whereas it is -5,20,60, 80°C after in-jection molding			Water uptake capacity is higher for SPI/TEG 621% and lower for SPI/EG-255%
**5h**			Highest at 90°C-1.52 (0% SB) and 0.99 (1%SB)	Max at 130°C-1.06 (0% SB) and 0.99 (1%SB).	decom-position peak SB-150°C, SPI/GL-305°C, Gly-230°C	Max at 90°C-15.3, min at 130°C-6.8		WUC-1700% with 0%SB, Max of 2250 and 2500% with 1% SB or SC
**6a**	control-(22.52± 0.43) Min with UV ex-posure at 24hr-(6.96± 0.60) ×106 g cm/m²d ay mm Hg)	Control-(105.31±2 .98), Min with heat curing at 70°C-50.32 ±1.09 (g cm/ m² day mmHg)	Control-0.557±08, Max with for-maldeyde-1.85±0.2, glutaraldehyde (1.98±0.4)	Control-(140.73±26.63) , Max with UV exposure for 24hr-160.34 ±39.79 and heat cur-ing at 80°C -98.26±32.25		Control-(1.79±0.31), Max with glu-taralde-hyde (6.18±0.78), formal-dehyde (5.76±0.56)	Control-(49.99±1.18), Succinic anhy-dride-(57.24±1.82)	
**6b**			Max at 3%GNP-(22.01), Min at 5%GNP-(16.07) LDPE:5.90	Max at 3%GNP-(89.82%), Min at 5%GNP-(64.73%) LDPE:291.66		Max at 5% GNP-(136.10), Min at 3% GNP-(111.50)		

						LDPE:218.33	
**6c**					Weight loss%-mix 2-7.6, mix3-1.9, pean-mat-6.5		
**6d**		PS/PPI Min (60:40)-0.00017and max (100:0)-0.00046	PPI (0 to 50%): pea starch reduced from 5.44 to 3.06.	PPI (0 to 50%): pea starch increased from 28.56 to 98.12%			Max at 100 PS / 0 PPI-22.31, Min at 50PS/50PPI-9.78±0.12
**7a**		Min at 0%GSE-0.023 Max at 1.5% GSE-0.033	Max with 0% GSE - 31.87± 4.44, Min at1.5% GSE-11.93± 0.9	Min at 0%GSE-13.37±3.40, Max at 1.5%GSE-72.74±5.5			
**7b**	OTR More than 2000 [$cm^3\cdot mm/(m^2\cdot day\cdot atm)$]		RC had the lowest stress and 5wt.% siccative became brittle	RC lowest compared to CR and CA		RC and CA have similar value half of CR	
**7c**			0%RO-4.9, 1%-4.3, 2%-5.9, 3%-6.3	0%RO-34.4, 1%- 43.7, 2%-45.9, 3%-73.1		0%RO-23.6, 1%-16.9, 2%-20.1, 3%-31.5	0% RO-42.4, 1%-40.9, 2%-40.7,3%-37.4
**7d**		Max (3% RP /5%G)-0.031	Max (3%RP/ 3,4%G)-53.45 ±3.6, Min (6%RP/ 4%G) 0.50±0.66	Max (5% RP/ 0.7% GC)-54.45 ± 2.24% and (2%RP /0.5% GC)-52.61±6.01, Min (6%RP/4%G) 2.10±1.01			
**7e**		Max for RPI, decrease gradually with CH and increase mildly with increase in	Min for RPI, gradually inc with inc in % hydrolysis, max at 12%RPH-CH (1:1)	RPI-CH had min value reached max with inc in % hydrolysis and 12%RPH-CH is greatest (1:1 ratio)			

	% hydrolysis at 1:1 of RPI/CH						
**7f**		Increases with inc in mold T, more evident in M-RM, MS-RM	Increases with in mold T, more evident in M-RM, MS-RM			Loss of soluble matter 52.7-54.3	Highest of 186.7±1.6 for M-RM at 80°C
**7g**	RPI-0.032, 5%SRPI-0.013,15 % SRPI-0.041	RPI-3.42, 5%SRPI-5.02 15%SRPI-2.35	RPI-84.17, 5%SRPI-123.6 15% SRPI-76.22	5% SRPI have best thermal stability with Tm of 95.46,243.34, 316.76°C at region I, II, III			5% SRPI-108.68º, 10& 15% SRPI <65º, RPI-77.66º,
**8a**		Highest for extruded WG/GL/W at pH 9	Highest for extruded WG/GL/W at pH 9		Highest for WG/Gly/W, Lowest for WG/GL/GXAL-W		Additives gave limited swellability, retaining a lower amount of water.
**8b**		Control-(2.6 ± 0.2) ,1(2.2±0.3), 2(2.9 ±0.4), 3(1.7± 0.22), 4(1.4±0.3),5(3.8±0.9),6(2.8±0.4)	Control-(237.9 ±21.9),1(267.2 ± .01), 2(192.3 ± 24. 9), 3(313.5 ±3 4.5) ,4(417.0± 41.5),5 (162.2± 40.3),6 (215.0±30.3)				
**8c**	WG-air 15.8, support 17.3; EWG air, support-7.2 WVTR (10^{-3} g m^{-2} s^{-1})	Decrease as aw increase for both WG and EWG (lower)	Increase as aw increases for both WG and EWG (higher)	Tg of pure gluten-162°C.Tg less than 25°C at aw>0.43. Tg decreased as aw increased	Decrease in value as a_w increase from 0.1-0.9 in WG and EWG (lower) films		Contact angle-WG air-35.7, WG support-53.6, EWG air-64.3, support-65.2

**8d**			WG-3.40, Max 7.51 at 2.5 wt.% FS and value dec on inc conc	WG-119%, WG+ FS -brittle				
**8e**			Min at 25°C-0.52 and max at 125°C-6.69	Min at 25°C-173.7 and max at 105°C-288.5 and decreases at 125°C-240.3	Change in molding temperature had no Influence on Tg	Min at 25°C-1.2 and max at 125°C-36.0		
**8f**			WG-3.40, SSP max at 2.5wt.% -6.53 and 10wt.% -3.78, CSSP-2.5wt.%- 16.50	WG-118.99, SSP at 2.5wt.% -62.83 and at 10wt.% -42.89, CSSP-12.74	Tmax of WG-322°C, 2.5%SSP -315°C, 2.5% CSSP-334°C			
**8g**	Gas permeability WG/TP (O_2-49689, CO_2:59 000), WG/UTP (O_2-8328, CO_2-16927) (10^{-18} mol $m^{-1}s^{-1}$ Pa^{-1})	TP-5.91, UTP-3.0, WG/TP-3.2, WG/UTP -1.27 (10^{-11} mol $m^{-1}s^{-1}$ Pa^{-1})						Contact angle WG: TP-76.3°, WG: UTP-15.7°; water: TP-109.7°, water: UTP-43.3°
**8h**							Reference system with water (25%), both sugar & water-(<50%), with sugar & no water (>50%)	Water absorption highest for 20% trehalose with (no water-WGP /GLY)-750%, same composition with water-380%. water (>50%)

**8i**		20.1-20.3 WVTR (g h^{-1}m^{-2})	2.6-6.5 with glycerol and 3.3-4.9 with 1,4butanediol with increasing filler	120 to 29.8 with glycerol and from 105.2 to 22.2 with 1,4 butanediol as a plasticizer and increasing filler				41 to 22° for glycerol, 32-29° for 1,4-butanediol with increasing filler
**9a**	SPI (0.6±0.03), CZ /SPI (0.81±0.05), ny/LLDPE (3.51±0.08) [*10^{-18}m^3 m /m^2 s Pa]	SPI (0.94±0.06), CZ/SPI (0.61±0.05), ny/LLDPE (<0.001) [* 10^{-12}kg m /m^2 s Pa]	SPI (2.5±0.5), CZ/SPI (5.9±0.4), ny/LLDPE (31.6±6.5)	SPI (178.6± 19.7), CZ/SPI (7.3± 2.0), ny/LLDPE (179.2±25.9)			CZ/SPI is 50% soluble at 90ºC	
**9b**		Z+Oa(0.51±0.07), Z+B-(0.82 ±.0.09), Z+M (0.76±0.05), Z+Oo(0.45±0.02)					Z+Oa (5.04±1.58),Z+B- (14.34±.4.19), Z+M(14.34±4.19),Z+Oo (23.19±2.85)	Films with oleic acid were only hydrophobic. Others are hydrophilic
**9c**		Control:0.377 , MCT oil-0.345, other wax/ oil coated-0.004	Control-1.05 ± 0.09, max: MCT oil 1.54± 0.05, Min: CW/MCT oil 0.82 ±0.02	Control-130.2 ±6.9, max: wax/oil ratios of 167.9±9.1				
**10**	Film-5.63, Amaranth flour-0.00065 × 1010 (cm^{3}m^{-1} s^{-1}Pa^{-1})	0.387×10-3 Amaranth flour-0.025 ×1010 (gm^{-1} s^{-1} Pa^{-1})	1.45 ± 0.04	83.74 ± 5.11	Tg- 76-85	215.0 ± 1.4	42.25±1.82	
**11a**		SI (1.45±0.01), SIw(1.49±0.07)SIg(1.46±0.01) (g H_2O Pa^{-1} s^{-1}m^{-1})	All-4	All-24	Tg_1 (69°C) glycerol-rich phase & Tg_2 (-28°C) protein-rich phase	0.58-all protein isolate films	SI- 40%, SIw & SIg -more soluble in water and all in buffer solutions	SI (50±0.75), SIw (70.0±1.39), SIg(66.69 ±0.80)

**11b**		3.2	73		17.7		Swelling capacity-180%
**11C**		Control-2.8, Max: (3% glyoxal -5.2, 6% tara tannin-4.4, 3% octanoic acid-7.	Control-37.6%, 2% glyoxal slightly increase & 3.5% Glutaraldehyde dec by 40%, Chestnut tannin- 28%, 4% octanol inc by 2%,			All additives are insoluble in water due to plasticization	Water uptake capacity (%): control-32, 0.7% glutaraldehyde-15, 3.2% glyoxal-22 ; contact angle: control-17.9°, 3.3% HCHO-42°, 0.6% decanol-95°
**11d**		Max with LiOH, NaOH-3.9, least NH_4OH-1.3, different plasticizers (1.8-27.1)	Maxi with NaOH base-251, least NH_4OH-86, different plasticizers (1.6-251)			68% for 2mins, 89% for 10 mins and 92% for 12mins.	
**12a**		Max:RPC/Gly (70/30) Sodium Bisulfite, Min: RPC/Gly (70/30) glyoxal	Max: RPC/Gly (70/30) Cyst, Min RPC/Gly (70/30)	RPC-96C, RPC-GL blends between 70-90C	Max: RPC/Gly (70/30), Min RPC/Gly (70/30) GLX		
**12b**				Tg: 60-70C			Water absorption <50% and least with 33wt.% glycerol 20%.
**12c**		Max at 150°C-0.26, 130°C-0.18 and min at 100°C- 0.12,	Max at 150°C-2.4, 130°C-1.6 and min at 100°C-1.1,		Max at 150°C- 33 and 130°C-23; Min at 100°C-12	Soluble matter loss from 32.2 to 27.3 for 100 to 150°C	The water uptake capacity-254 to 137% for 100 to 150°C
**13**	5.5±0.2	2.1±0.3	106.1±9.7				Karafin has total hydrophobic amino acid of 58.3%

**14a**	CP + glutaraldehyde had the highest micro hardness of 45MPa		Td of CP decreases from 156.2 °C to 136.0 °C when the glycerol increases from 10 to 30 wt.%, respectively		CP-FA, CP-GX, and CP-GA absorbed less water than the standard sample (CP-0CL)
**14b**	CP-GA-45, CP-OCL-2		High thermal stability: CP-FA had least mass loss<190 C, CP-GA >190C,		water absorption (%) CP-0CL 40.90, CP-FA-37.23%, CP-GX-33.27, CP-GA-32.43
**14c**	C1- 0.48 (PCL-100, WCSM-0), X1-0.34(PCL-100, CSO-20), XC3-0.24 (PCL-80, CPI-20, CSO-20), XS3-0.17 (PCL-80, SPI-20, CSO-20),	MAX:C1-42(PCL-100, WCSM-0), X3-14 (PCL-80, WCSM-20, CSO-20), XC3-10 (PCL-80, CPI-20, CSO-20), xs3-17(PCL-80, SPI-20, CSO-20)	For PCL /WCSM / CO-degraded at 250-380C, X1(PCL/CO 100:20) degraded at 350-420C, WCSM degrades by itself and not interacting with PCL/CO	C5-13.1 (PCL-60, WCSM-40), X3-9.86 (PCL-80, WCSM - 20, CSO-20), XC5 - 11 (PCL-60, CPI-20, CSO-20, CPI-40), XS5-6.34 (PCL-60, SPI-40, CSO-20)	
